# COST-EFFECTIVENESS ANALYSIS OF THE COLLABORATIVE STEPPED CARE INTERVENTION FOR LATE-LIFE DEPRESSION

**DOI:** 10.1093/geroni/igz038.3213

**Published:** 2019-11-08

**Authors:** Shiyu Lu, Gloria H Y Wong, Terry Lum, Tianyin Liu

**Affiliations:** 1 The University of Hong Kong, Hong Kong, Hong Kong; 2 Department of Social Work and Social Administration, PokFuLam, Hong Kong

## Abstract

Late-life depression is a burden on society because it is costly and have a significant adverse effect on the quality of life. The aim of this study is to evaluate the cost-effectiveness of the collaborative stepped care intervention for depression among community-dwelling older adults compared to care as usual from a societal perspective. The intervention was piloted from 2016-2019 in Hong Kong. The study used a two-armed quasi-experimental design. Eventually, 412 older people were included (314 collaborative stepped care, 98 care as usual). Baseline measures and 12-month follow-up measures were assessed using questionnaires. We applied the 5-level EQ-5D version (EQ-5D-5L) and the Client Service Receipt Inventory (CSRI) respectively measuring quality-adjusted life-year (QALY) and health care utilization. The average annual direct medical cost in the intervention group was USD 6,589 (95% C.I., 4,979 to 8,199) compared to US$ 6,167 (95% C.I., 3,702 to 8,631) in the care as usual group. The average QALYs gained was 0.036 higher in the collaborative stepped care group, leading to an incremental cost-effectiveness ratio (ICER) of US$ 11,722 per QALY, lower than the cost-effectiveness threshold suggested by The National Institute for Health and Clinical Excellence. The study showed that collaborative stepped care was a cost-effective intervention for late-life depression over service as usual.

